# Graefe’s archive for clinical and experimental surgery—170th anniversary

**DOI:** 10.1007/s00417-023-06358-w

**Published:** 2024-01-17

**Authors:** Antonia M. Joussen

**Affiliations:** Department of Ophthalmology, Charité Universitätsmedizin Berlin, Hindenburgdamm 30, 12200 Berlin, Germany

In 1854, von Graefe founded the “Archive für Augenheilkunde” in Berlin. He established this journal with a mission to advance ophthalmic knowledge. After von Graefe’s untimely death in 1870, co-editors and friends Ferdinand von Arlt from Vienna and Frans Cornelius Donders from Utrecht/Netherlands renamed the journal in his honor to “Graefe’s Archive for Ophthalmology” thus solidifying Graefe’s enduring impact on the field.

Albrecht von Graefe (1828–1870) stands out as a pioneering personality in the history of ophthalmology, making indelible contributions that have shaped the field into what it is today. As the founder of the journal and a visionary clinician and scientist, von Graefe’s legacy extends beyond his groundbreaking discoveries in all areas of ophthalmology (e.g., glaucoma, central artery occlusion, and tuberculosis). His impact on modern ophthalmology was not only by his scientific discoveries, but also by his exceptional personality encompassing internationalism, friendship, self-criticism, love of truth, and modesty. His enduring legacy is characterized by the internationalization of the field, the establishment of renowned institutions, and the recognition of excellence through prestigious awards.

With this in mind, we acknowledge that *Graefe’s Archive of Ophthalmology* is the first truly international journal in the field.

But how can the value of a journal endure over such a long time? 170 years of scientific reports from ophthalmology—this requires flexibility and continuous adjustments in the way of scientific reporting as much as clinical practice and treatment processes constantly improve with scientific insights and technical developments.

I met Diana Epstein as the editorial manager of *Graefe’s Archives* in 2001 when she was working in our Department in Cologne while Günther Krieglstein was the Editor-in-Chief. When my mentor Bernd Kirchhof, took over as Editor-in-Chief the transition from a printed review process to online began—this process was quicker and more efficient than the process of digitalization which took place in the clinics.

Since 2011, I have had the pleasure to serve as Editor-in-Chief of *Graefe’s Archives*. And as during the times of Graefe, Arlt, and Donders, I had the chance at that time to share the Editor-in-Chief job with a great colleague and friend, David Wong, (2011–2018); we became an international team that has always been supported by an international editorial board to ensure *Graefe’s Archives of Ophthalmology* had the cover and competence in all areas of Ophthalmology.

Since 2019, the role of Editor-in-Chief has been shared between myself, Vas Sadda, and Taiji Sakamoto, again compiling a truly international team from Europe, the USA, and Asia. The three of us are Retina experts, hence with the broad scientific knowledge of the editorial board and with the assistance of Diana Epstein as head of the editorial office, we are able to balance the topics.

Why were the broad scientific spectrum and the internationality already so much in the scope 170 years ago when *Graefe’s Archives of Ophthalmology* was founded? Albrecht von Graefe addressed almost all facets of ophthalmology, with ophthalmopathology and general disease relationships being particularly important for him. Although he considered himself a “specialist in ophthalmology”, he always saw ophthalmology as part of all medicine. Albrecht von Graefe was always in search of the “scientific truth”. He was always questioning himself, often correcting himself. The scientific discussion was important to him. He had numerous international friends beyond Arlt and Donders, just to mention two of the well-known ophthalmologists, William Bowman (London), and Friedrich Horner (Zürich). Thanks to Graefe, the congresses of the DOG (German Society of Ophthalmology) in Heidelberg were visited by international colleagues since its founding. Graefe had many patients from abroad and founded our “*Graefe’s Archives*” to enhance the international scientific discourse.

We hope to continue this path in the future.

One could, however, ask, if for that international scientific discourse, a scientific journal is still needed. In today’s age of artificial intelligence and ChatGPT, are we still in need of journals such as *Graefe Archives*—although in electronic versions?

Yes! Scientific progress will only be achieved when thorough clinical observations and experimental proof of concepts are published with a discussion and compiled in scientific articles as provided by journals like *Graefe’s Archives*. It is along these lines of Albrecht von Graefe, who compiled his clinical observations and reasonings with a discussion in a broader and already international community when he founded his Archives. And these two integral parts: the observation or experimental assessment, and secondly the critical reasoning on the base of a discussion amongst experts. Even in the future, this will remain a “conditio sine qua non”, and there is no scientific progress without these two parts coming together. Digital applications will improve healthcare and patient care in the future. To this end, artificial intelligence will help to analyze and integrate big data sets. It will also influence the scientific use of data coming from “omics” analyses and improvements in automated imaging assessments. Artificial intelligence will accelerate the extractability and usability of data. It will however not replace the scientific discourse in a journal like *Graefe Archives.*

Still, a lot of tasks remain for *Graefe’s Archive* as a scientific journal.

What are the topics for the future? The aging population presents a unique challenge in ophthalmology, with simulations predicting a 50% increase in patients requiring treatment for age-related retinal diseases, also while diabetes care is progressing (e.g., thanks to AI in BP control), the worldwide numbers of patients affected with diabetes associated eye disease is increasing worldwide. There is a high demand for care in childhood ocular diseases. This area is in many countries vastly underfunded. Scientific topics might change in the future, but we need to maintain the broadness of the spectrum in ophthalmology and ophthalmic science, as was the intention of Albrecht von Graefe when he founded the *Archives of Ophthalmology*.

In 1882, on his 54th birthday, the Graefe monument at the Charité was unveiled and inaugurated. It was the first monument for a scientist in Berlin, donated by colleagues and friends from all over the world, including the Russian Tsar. The monument is located in Berlin in front of the entrance of the Charité Campus Mitte.

Von Graefe’s relationship with the Charité in Berlin, where he later became the chair of ophthalmology, was marked by challenges. In particular, in his letters to his pupil and friend Julius Jacobson in Königsberg (now Kaliningrad, Russia), Graefe complained bitterly about the lack of equipment, the faculty, and the Prussian ministerial bureaucracy. Despite this, his private clinic, the DOG (German Society of Ophthalmology), and his archive served as vital counterpoles to institutional challenges.

Things have not changed in the past 170 years.

Albrecht von Graefe vehemently advocated for the separation of ophthalmology at all universities. However, he did not live to see this fulfilled. If today we are independent ophthalmologists and not appendices of surgery, that is in no small way due to Albrecht von Graefe. His friend, the famous pathologist at the Charité Rudolf Virchow, was against the independence of ophthalmology.

In several counties, eye clinics are increasingly owned by private equity investors. This puts the focus of these clinics on the most profitable areas of ophthalmology and the expansion of volume in selected fields. At the same time, publicly funded academic eye centers are being left with complex, financially unattractive cases, and loose market share. This imbalance puts science in ophthalmology in jeopardy. Like 170 years ago, ophthalmology has to continue being an integral part of academic medicine to maintain its independence and leverage its full innovation potential.

In Europe, but also all over the world, the medical systems currently face a lot of changes. This affects science; this will affect science reporting, and thus the journals.

**Fig. 1:**
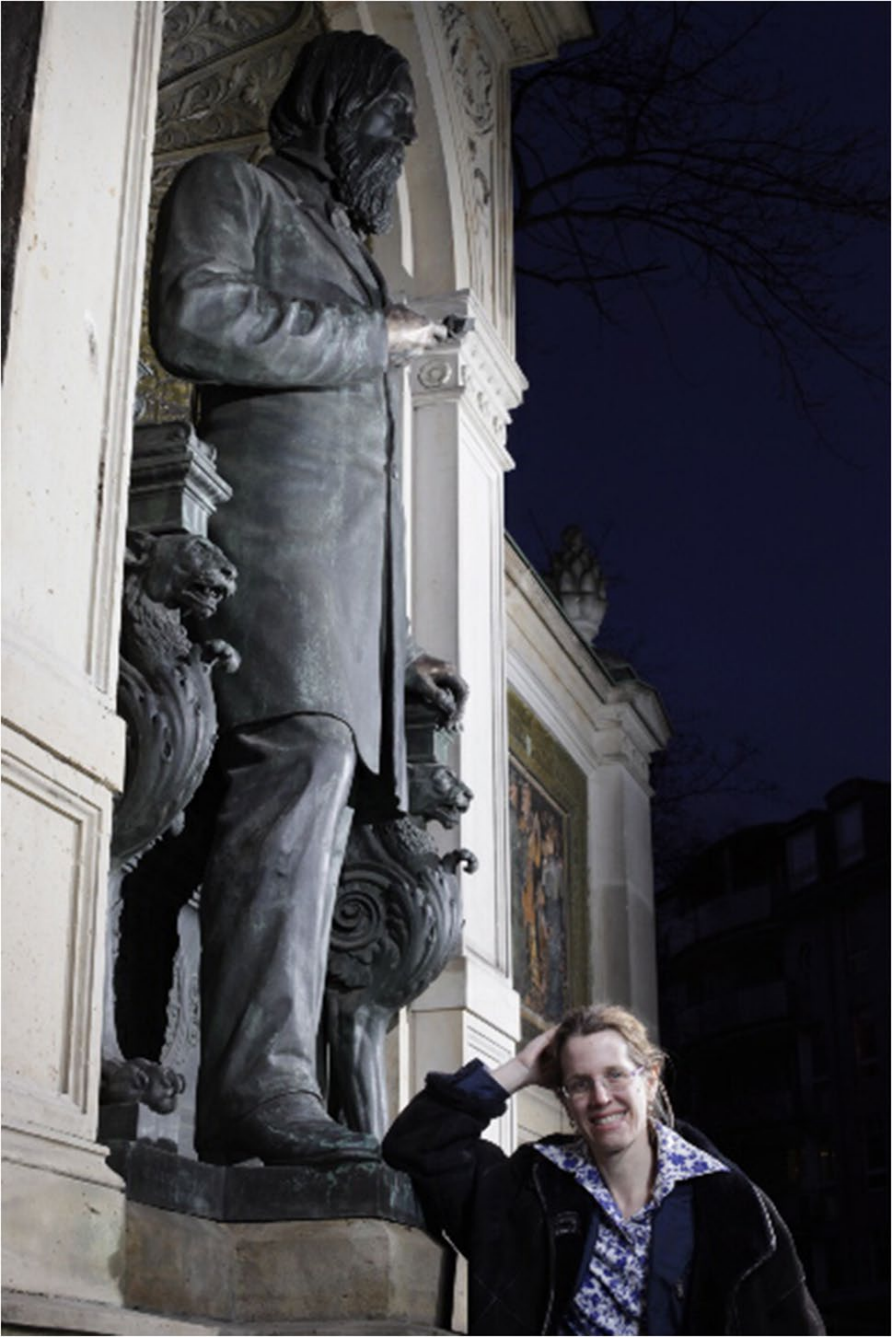
Joussen 2010 in front of the Graefes Monument at the Charité Central Campus, in Berlin Germany. Prof. Joussen is Chair of the Department of Ophthalmology at the Charité University Medicine Berlin since 2010 and has amalgamated the two departments of the former Humboldt University and the Freie University Berlin under her leadership

It is important that we learn from our founder whose purpose was primarily to help other people, irrespective of their origin or income. Graefe was fully aware of the importance of sight for humans. Keeping this in mind, I wish our journal prosperity and wise choices in maintaining the scientific discourse in ophthalmology (Fig. 1).

